# Rapid establishment of a frontline field laboratory in response to an imported outbreak of Ebola virus disease in western Uganda, June 2019

**DOI:** 10.1371/journal.pntd.0009967

**Published:** 2021-12-03

**Authors:** Amy J. Schuh, Jackson Kyondo, James Graziano, Stephen Balinandi, Markus H. Kainulainen, Alex Tumusiime, Luke Nyakarahuka, Sophia Mulei, Jimmy Baluku, William Lonergan, Oren Mayer, Rastus Masereka, Fredrick Masereka, Esther Businge, Alphonse Gatare, Loice Kabyanga, Samuel Muhindo, Raymond Mugabe, Issa Makumbi, Joshua Kayiwa, Milton Makoba Wetaka, Vance Brown, Joseph Ojwang, Lisa Nelson, Monica Millard, Stuart T. Nichol, Joel M. Montgomery, Celine H. Taboy, Julius J. Lutwama, John D. Klena

**Affiliations:** 1 Viral Special Pathogens Branch, Division of High-Consequence Pathogens and Pathology, United States Centers for Disease Control and Prevention, Atlanta, Georgia, United States of America; 2 United States Public Health Service Commissioned Corps, Rockville, Maryland, United States of America; 3 Department of Arbovirology, Emerging and Reemerging Infectious Diseases, Uganda Virus Research Institute, Entebbe, Uganda; 4 Bwera General Hospital, Bwera, Uganda; 5 Kasese District Health Office, Kasese, Uganda; 6 Uganda Central Public Health Laboratories, Kampala, Uganda; 7 Uganda Public Health Emergency Operations Center, Kampala, Uganda; 8 United States Centers for Disease Control and Prevention, Kampala, Uganda; 9 Walter Reed Army Institute of Research, Kampala, Uganda; University of Texas Medical Branch, UNITED STATES

## Abstract

The Democratic Republic of the Congo (DRC) declared an Ebola virus disease (EVD) outbreak in North Kivu in August 2018. By June 2019, the outbreak had spread to 26 health zones in northeastern DRC, causing >2,000 reported cases and >1,000 deaths. On June 10, 2019, three members of a Congolese family with EVD-like symptoms traveled to western Uganda’s Kasese District to seek medical care. Shortly thereafter, the Viral Hemorrhagic Fever Surveillance and Laboratory Program (VHF program) at the Uganda Virus Research Institute (UVRI) confirmed that all three patients had EVD. The Ugandan Ministry of Health declared an outbreak of EVD in Uganda’s Kasese District, notified the World Health Organization, and initiated a rapid response to contain the outbreak. As part of this response, UVRI and the United States Centers for Disease Control and Prevention, with the support of Uganda’s Public Health Emergency Operations Center, the Kasese District Health Team, the Superintendent of Bwera General Hospital, the United States Department of Defense’s Makerere University Walter Reed Project, and the United States Mission to Kampala’s Global Health Security Technical Working Group, jointly established an Ebola Field Laboratory in Kasese District at Bwera General Hospital, proximal to an Ebola Treatment Unit (ETU). The laboratory consisted of a rapid containment kit for viral inactivation of patient specimens and a GeneXpert Instrument for performing Xpert Ebola assays. Laboratory staff tested 76 specimens from alert and suspect cases of EVD; the majority were admitted to the ETU (89.3%) and reported recent travel to the DRC (58.9%). Although no EVD cases were detected by the field laboratory, it played an important role in patient management and epidemiological surveillance by providing diagnostic results in <3 hours. The integration of the field laboratory into Uganda’s National VHF Program also enabled patient specimens to be referred to Entebbe for confirmatory EBOV testing and testing for other hemorrhagic fever viruses that circulate in Uganda.

## Author summary

Following an imported outbreak of Ebola virus disease in Uganda’s western Kasese District, the Uganda Virus Research Institute and the United States Centers for Disease Control and Prevention jointly established a frontline field laboratory to test specimens collected from alert and suspect cases for Ebola virus disease. Using a single room equipped with a rapid containment kit to safely inactivate patient specimens and a GeneXpert to perform the Xpert Ebola Assay, the field laboratory rapidly ruled-out Ebola virus disease as the cause of illness in 76 patients during its 46 operational days. All specimens were also referred to Uganda Virus Research Institute (Entebbe) for confirmatory Ebola virus testing and testing against a panel of viruses known to cause hemorrhagic fever in Uganda, in line with the National Viral Hemorrhagic Fever Program’s testing protocol and mandate. The Ebola field laboratory served as a valuable asset in the outbreak response by supporting patient management and epidemiological surveillance.

## Introduction

Following spillover from an unidentified zoonotic source, such as a bat, non-human primate, or other animal sources (e.g., duiker), Ebola virus (EBOV; family *Filoviridae*, genus *Ebolavirus*, species *Zaire ebolavirus*) can be spread from person-to-person, resulting in large outbreaks of Ebola virus disease (EVD) with high case fatality [1]. On August 1, 2018, the Ministry of Health (MoH) of the Democratic Republic of the Congo (DRC) declared the country’s 10^th^ outbreak of EVD in the northeastern province of North Kivu [2,3]. By June 10, 2019, EVD had been detected in 26 health zones within DRC’s North Kivu and Ituri Provinces, and caused a reported 2,071 cases (1,977 confirmed and 94 probable) and 1,396 deaths [4]. Due to the high frequency of population movements across national borders for trade, social events and asylum, the WHO categorized the regional risk of EVD spillover to DRCs neighboring countries of Uganda, Rwanda and South Sudan as very high [5].

On June 10, 2019, a mother from Masambu Village in DRC’s North Kivu Province traveled with her sick 5-year old child across the Mpondwe Border Post to seek medical care at Kagando Hospital in Uganda’s western Kasese District (Table 1) [6]. Healthcare workers suspected EVD as the cause of the 5-year old’s illness, as the child presented with bleeding diathesis and a family member had alerted the hospital that the child may have been infected with EBOV. The same evening, prior to transferring the 5-year old by ambulance to the Ebola Treatment Unit (ETU) at Bwera General Hospital, a blood specimen collected at Kagando Hospital was sent to the Viral Hemorrhagic Fever (VHF) Surveillance and Laboratory Program at the Uganda Virus Research Institute (UVRI) in Entebbe, Uganda to test for a panel of hemorrhagic fever viruses, including ebolavirus (EBOV, Sudan virus [SUDV], and Bundibugyo virus [BDBV]), marburgvirus (Marburg virus [MARV] and Ravn virus [RAVV]), Crimean Congo hemorrhagic fever virus (CCHFV) and Rift Valley fever virus (RVFV). Test results released by UVRI the next morning (June 11) confirmed that the 5-year-old was positive for EBOV; the child died later that night. The Ugandan MoH formally declared the EVD outbreak in Kasese District the evening of June 11 and notified the World Health Organization (WHO) [4]. Concurrently on June 10, the 50-year old grandmother and 3-year old brother of the 5-year old child entered Uganda from the DRC and were admitted to the ETU at Bwera General Hospital [6]. Blood specimens collected from these two relatives on the night of June 11 also tested positive for EBOV at UVRI the morning of June 12; the 50-year-old grandmother and 3-year-old child succumbed to EVD on June 12 and 13, respectively.

**Table 1:** Timeline of key events in the June 2019 imported outbreak of Ebola virus disease in Uganda and establishment and operation of the Uganda Virus Research Institute-United States Centers for Disease Control and Prevention Ebola Field Laboratory at Bwera General Hospital.

Date (time)	Event
Jun 11 (AM)	5-year-old child tests positive for EBOV
Jun 11 (PM)	Ugandan MoH formally declares an outbreak of EVD in Kasese District and notifies the WHO
Jun 11 (PM)	5-year-old child dies
Jun 12 (AM)	50-year-old grandmother to 5-year-old child tests positive for EBOV
Jun 12 (AM)	3-year-old brother to 5-year-old child tests positive for EBOV
Jun 12	50-year-old grandmother dies
Jun 13	Repatriated 3-year-old child dies
Jun 14	Ugandan Minister of Health and DGHS formally request that UVRI and CDC jointly establish an EBOV field laboratory in Kasese District
Jun 16	Deployment of laboratorians, equipment, and supplies to the Kasese District
Jun 17	Meetings with Kasese DHT and Bwera General Hospital leadership; assessment of a potential laboratory site at Bwera General Hospital
Jun 18	Site of UVRI-CDC Ebola Field Laboratory at Bwera General Hospital is cleaned and disinfected
Jun 18	Electrical and carpentry work for the laboratory is completed
Jun 20	Laboratory equipment is set-up
Jun 21	Xpert Ebola Assay quality control is successfully completed on the GeneXpert Instrument
Jun 22	UVRI-CDC Ebola Field Laboratory accepts its first specimen
Jul 4	Day 21 of the outbreak
Jul 25	Day 42 of the outbreak
Aug 7	Operation of the UVRI-CDC Ebola Field Laboratory is discontinued
Aug 9	9-year-old child tests positive for EBOV
Aug 31	UVRI-CDC Ebola Field Laboratory rapidly redeploys and begins testing patient specimens

EBOV, Ebola virus; EVD, Ebola virus disease; MoH, Ministry of Health; WHO, World Health Organization; DGHS, Director General of Health Services; Kasese District Health Team (DHT) UVRI, Uganda Virus Research Institute; CDC, United States Centers for Disease Control and Prevention.

**Fig 1:**
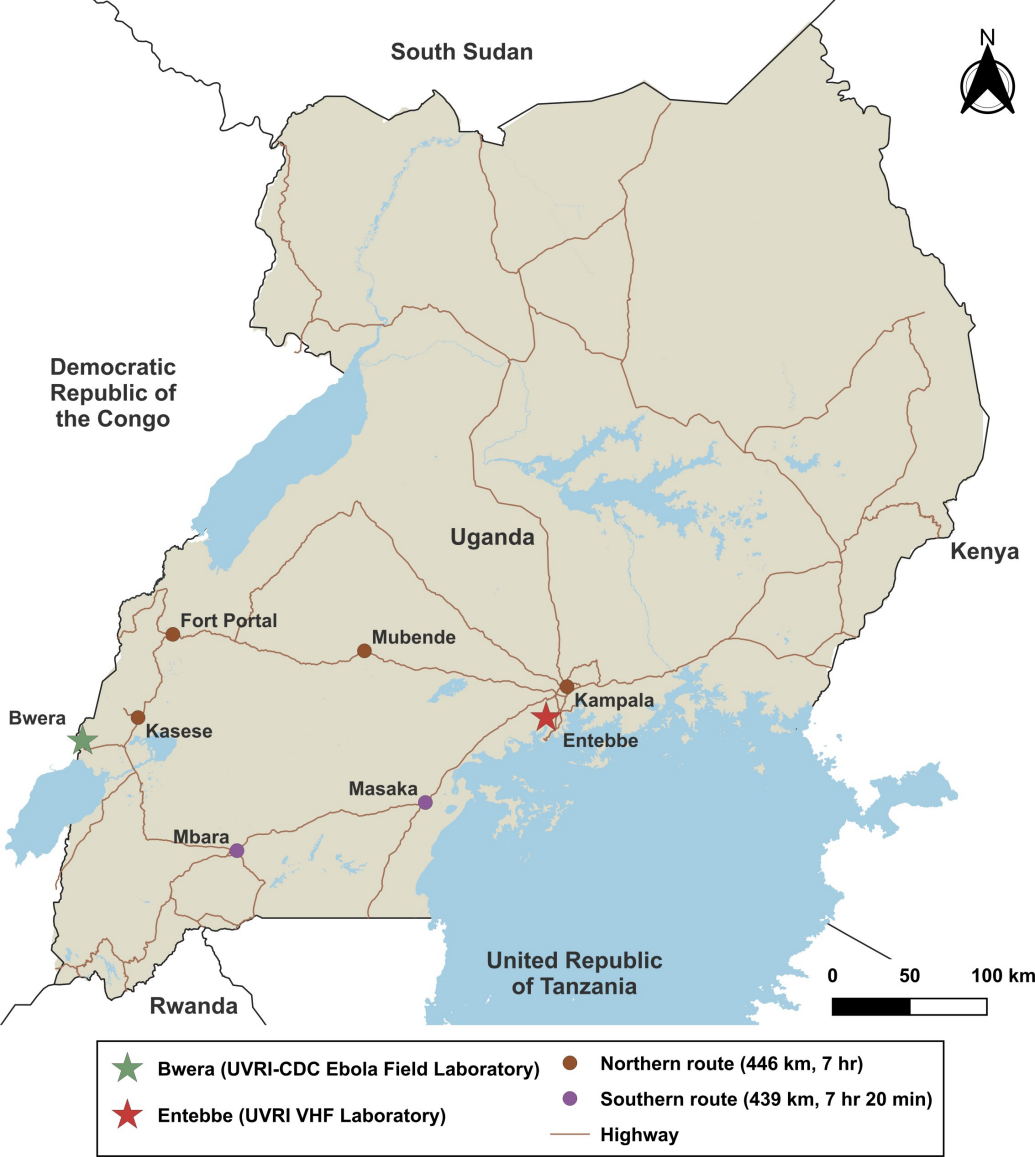
Map depicting the travel time to transport patient specimens by vehicle from the Uganda Virus Research Institute-United States Centers for Disease Control and Prevention Ebola Field Laboratory in Bwera to the UVRI Viral Hemorrhagic Fever Laboratory in Entebbe. The map was created using QGIS version 3.20.1 (https://www.qgis.org). The Uganda basemap (uga_admbnda_ubos_20200824_SHP.zipSHP) was obtained from The World Bank Data Catalog (https://data.humdata.org/dataset/uganda-administrative-boundaries-admin-1-admin-3?force_layout=desktop).

The Ugandan MoH, National Public Health Emergency Operating Center (PHEOC) and the Kasese District Health Team (DHT), with the support of additional national and international partners, initiated a rapid, multisectoral response to contain the outbreak [4]. As part of this response, the Ugandan Minister of Health and Director General of Health Services (DGHS), requested that UVRI and United States (US) Centers for Disease Control and Prevention (CDC) jointly establish a frontline field laboratory in the Kasese District (439–446 km or 7-8-hour drive from the National VHF Reference Laboratory in Entebbe; Fig 1) to rapidly screen diagnostic specimens from alert and suspect cases of EVD for EBOV. With the support of Uganda’s PHEOC, the Kasese DHT, the Superintendent of Bwera General Hospital and his staff, the US Department of Defense’s Makerere University Walter Reed Project (MUWRP), and the US Mission to Kampala’s Global Health Security Technical Working Group, UVRI and CDC established an Ebola Field Laboratory in Kasese District at Bwera General Hospital, proximal to an Ebola Treatment Unit (ETU). Herein, we describe the deployment, establishment, operations, and outcomes of the UVRI-CDC Ebola Field Laboratory at Bwera General Hospital in western Uganda.

## Methods

### Ethics statement

The UVRI-CDC Field Laboratory at Bwera General Hospital was established as part of the EVD Outbreak Response in Uganda and was approved by the Ugandan MoH. All EBOV testing was performed for clinical diagnostic purposes in support of the CDC’s public health response to the EVD outbreak in Uganda and, thus, was not subject to institutional review board requirements. All the Xpert Ebola assay results from the field laboratory were immediately released by UVRI to the PHEOC for dissemination to all relevant public health officials as per the National VHF Program protocols. All test results were verified by confirmatory testing at the VHF Surveillance and Laboratory Program at UVRI, which is the designated National Reference Laboratory for VHF testing in Uganda.

### Case definitions

EBOV diagnostic specimens submitted to the UVRI-CDC Ebola Field Laboratory were collected from alert and suspect cases of EVD. During this outbreak, the alert case definition for EVD was used by community and community-based volunteers, while the suspect case definition for EVD was used by mobile teams, health stations, and health centers (Table 2) [7]. For later reference, the suspect case definition for VHF was used by the VHF Surveillance and Laboratory Program at UVRI for routine VHF surveillance [8,9]. The Uganda Viral Hemorrhagic Fever Surveillance Project Suspect Case Report Form (CRF) was used by the health care team to record patient information, clinical signs and symptoms, patient/clinical status, epidemiological risk factors and exposure, and clinical specimen and laboratory testing information.

**Table 2:** Ebola virus disease and viral hemorrhagic fever case definitions and descriptions.

Case Definition	Description
Alert case of EVD	Illness with onset of fever (≥38°C) and no response to treatment of usual causes of fever in the area, **OR** fever and recent travel to the DRC, **OR** at least one of the following signs: inexplicable bleeding, bloody diarrhea, bleeding into urine **OR** any sudden death.
Suspect case of EVD	Any person, alive or dead, suffering or having suffered from a sudden onset of high fever (≥38°C) and having had contact with a suspected, probable or confirmed EVD case or a dead or sick animal, **OR** any person with sudden onset of high fever and at least three of the following symptoms: headaches, anorexia/loss of appetite, lethargy, aching muscles or joints, breathing difficulties, vomiting, diarrhea, stomach pain, difficulty swallowing, hiccup, **OR** any person with inexplicable bleeding, **OR** any sudden, inexplicable death.
Suspect case of VHF	Any person with acute illness, fever ≥38°C and no alternative diagnosis (e.g., malaria) **AND** at least four of the following signs/symptoms: vomiting/nausea, diarrhea, muscle or joint pain, chills/rigors, abdominal pain, skin rash, difficulty swallowing, jaundice, intense fatigue, headache, or unexplained bleeding from any site.

EVD, Ebola virus disease; DRC, Democratic Republic of the Congo; VHF, Viral hemorrhagic fever.

### Specimen collection

**Fig 2:**
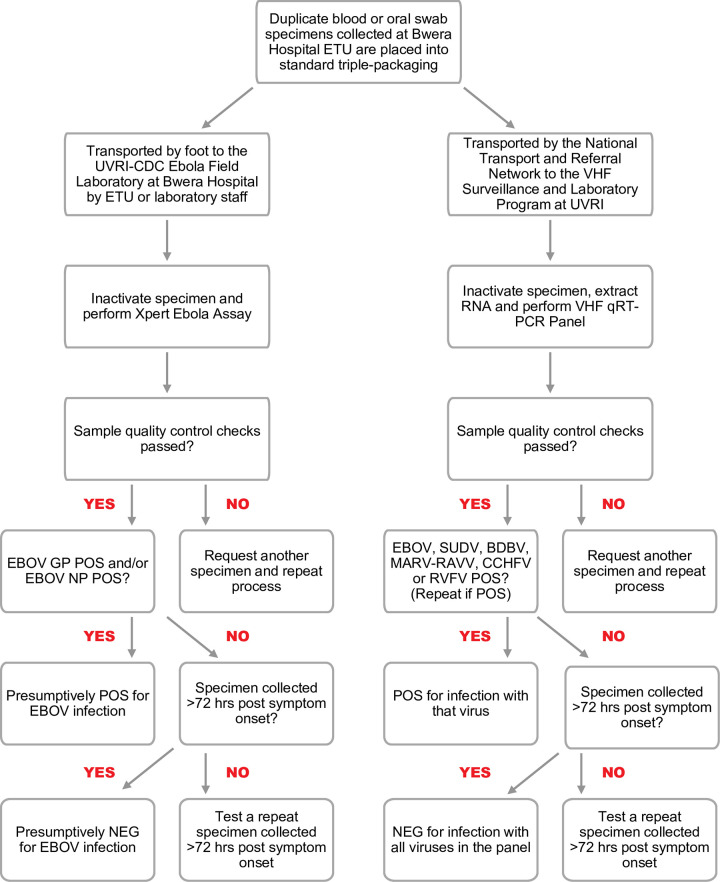
Specimen collection and diagnostic testing algorithm. ETU, Ebola Treatment Unit; UVRI, Uganda Virus Research Institute; CDC, United States Centers for Disease Control and Prevention; EBOV, Ebola virus; GP, Glycoprotein; NP, Nucleoprotein; NEG, Negative; RNA, Ribonucleic acid; VHF, Viral hemorrhagic fever; qRT-PCR, quantitative reverse transcriptase-polymerase chain reaction; SUDV, Sudan virus; BDBV, Bundibugyo virus; MARV-RAVN, Marburg virus-Ravn virus; CCHFV, Crimean Congo hemorrhagic fever virus; RVFV, Rift Valley fever virus.

Duplicate diagnostic specimens were collected by clinical personnel after donning the recommended personal protective equipment (PPE) [10]. Whole blood specimens were collected into EDTA vacutainer collection tubes and oral swabs were collected on occasion from corpses using cotton-tipped applicators (Fig 2). Prior to transport, each specimen was placed into standard triple-packaging (UN2814 Infectious Substance Triple Package, Exam Packaging, Strombeek-Bever, Belgium) [11]. One of the standard-triple packaged specimens was delivered to the UVRI-CDC Ebola Field Laboratory at Bwera General Hospital by ETU or laboratory staff, while the second specimen was transported by a WHO-hired vehicle via the National Transport and Referral Network in a cooler box containing an ice pack to the VHF Surveillance and Laboratory Program at UVRI for confirmatory EBOV testing (Fig 1), as well as testing for other hemorrhagic fever viruses found in Uganda including SUDV, BDBV, MARV, RAVV, RVFV and CCHFV. If the initial specimen was collected <72 hours after symptom onset and tested negative, the physician was advised to collect a repeat specimen 72 hours after the onset of symptoms to definitively rule-out EVD.

### Specimen receipt

**Fig 3:**
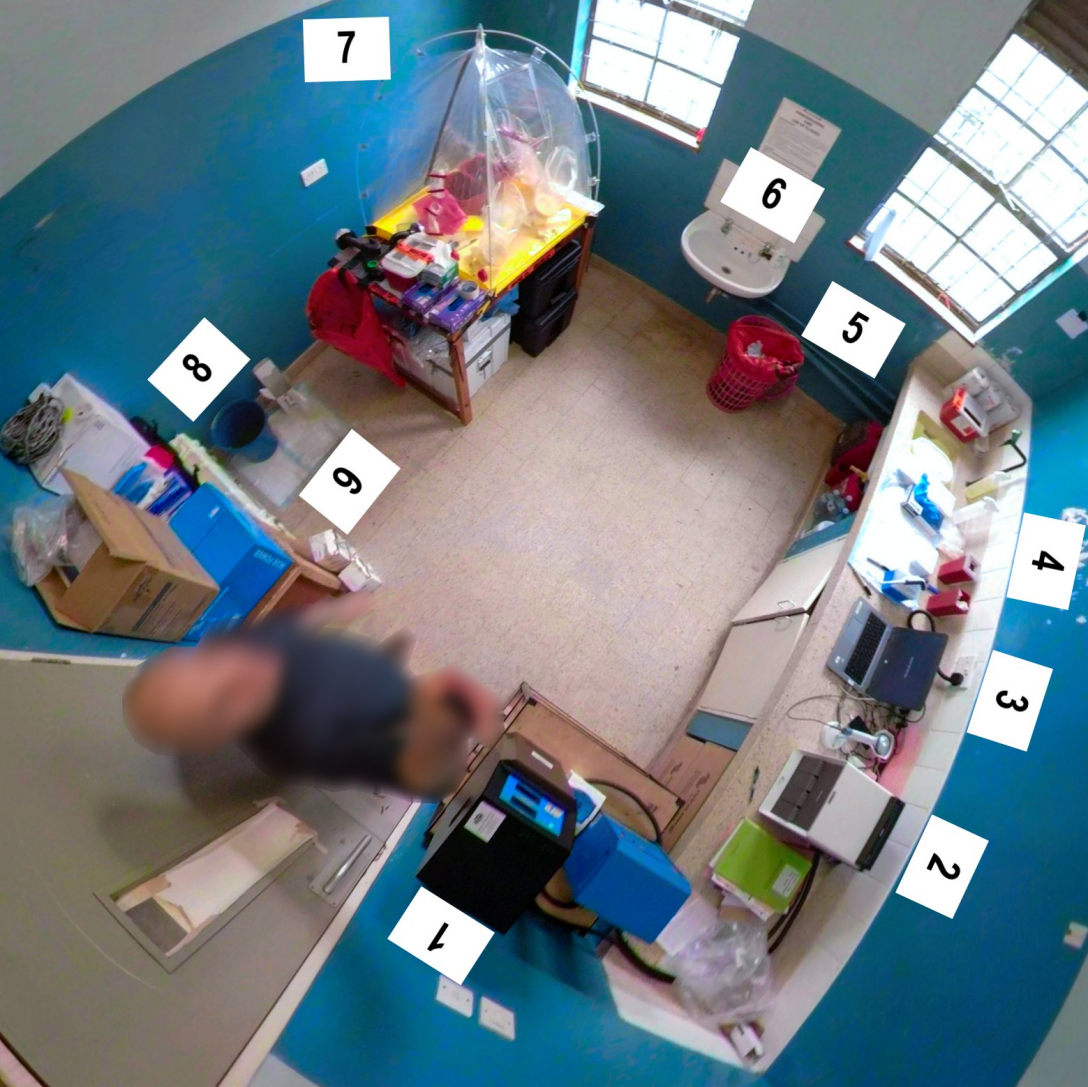
Fisheye view of the Uganda Virus Research Institute-United States Centers for Disease Control and Prevention Ebola Field Laboratory at Bwera General Hospital. Beginning at the door and moving counterclockwise, the major pieces of equipment and work areas that comprised the field laboratory included: 1) uninterruptable power supply with four large batteries, 2) GeneXpert Instrument, 3) GeneXpert-specific computer, 4) inactivated diagnostic specimen work area, 5) general laboratory waste, 6) sink for washing hands, 7) rapid containment kit used for specimen handling and viral inactivation of specimens, 8) designated area for decontamination of tertiary and secondary specimen transport containers, and 9) storage area for extra laboratory supplies.

Upon delivery of a diagnostic specimen to the UVRI-CDC Ebola Field Laboratory (Fig 3), a laboratorian donning procedure-specific PPE [this PPE included a gown (Sirus Surgical Level 3 Fabric Reinforced Gown, Medline, Northfield, IL, US), gloves and a face shield (Disposable Full Face Shield Anti-fog, Thermo Fisher Scientific, Waltman, MA, US)], used 5% Micro-Chem Plus Disinfectant Detergent (National Chemical Laboratories, Philadelphia, PA, US) to decontaminate the biohazard bag containing the triple-packaged specimen and CRF. The tertiary container and CRF were removed from the decontaminated biohazard bag and immediately decontaminated with 5% Micro-Chem. The secondary container was removed from the decontaminated tertiary container and immediately decontaminated with 5% Micro-Chem. Each specimen was assigned a unique identification number, and the CRF was used to record patient demographic information, signs and symptoms, as well as specimen time-stamp information.

### Rapid containment kit and PPE

As a biosafety precaution, all diagnostic specimens were processed within a rapid containment kit (RCK; Germfree Laboratories, Ormond Beach, FL, US). Two inlet/outlet ports of the RCK were fitted with high-efficiency particulate air (HEPA) filter cartridges and the glove ports were fitted with rugged, canner gloves (Canners and Handler Gloves, Unlined, 12” length, 20 mil, Ansell, Iselin, NJ, US). The RCK was maintained under negative pressure by a battery-operated, double HEPA-filtered exhaust air pump. Prior to each use, the entire surface area of the RCK and its gloves were inspected for tears and general wear while under negative pressure. Gloves were replaced at least once monthly as the first task of the day after all waste had been removed from the RCK and its interior surfaces had been decontaminated using the procedures described in detail below. Sufficient battery charge was checked by assessing the outflow of air pressure from the HEPA-filtered exhaust pump and visually ensuring that the RCK was slightly drawn inward from the negative air pressure. While working in the RCK, the laboratorian donned procedure-specific PPE, including a gown (Sirus Surgical Level 3 Fabric Reinforced Gown) and a double pair of gloves, with the inner pair taped to the gown. Following the completion of work in the RCK, the laboratorian removed their double-gloved hands from the inner RCK gloves. After the outer pair of gloves and gown sleeves were decontaminated with 5% Micro-Chem, the outer gloves were removed and placed into a large wastebin in the main laboratory, the inner gloves were decontaminated with 5% Micro-Chem, the tape was removed from the inner pair of gloves and placed into the wastebin, the gown was removed and hung-up for re-use, and the inner pair of gloves were removed and placed into the wastebin. The laboratorian then washed their hands with soap and water.

### Movement of items in and out of the RCK

The decontaminated secondary container was moved into the RCK by opening the outer airlock of the large bag-in/bag-out tube, placing the container into the tube, closing the outer airlock, opening the inner airlock, passing the container to the inside space, and then closing the inner airlock. After removing the Ziploc bag from the secondary container, the container and the laboratorian’s gloves were immediately surface decontaminated with 5% Micro-Chem. The decontaminated secondary container was removed from the RCK by opening the inner airlock of the large bag-in/bag-out tube, placing the decontaminated container into the tube, spraying the tube with 5% Micro-Chem, closing the inner airlock, opening the outer airlock after 3 min, removing the container from the tube, and then closing the outer airlock. The secondary container was immediately immersed in a bucket containing 5% Micro-Chem for a minimum exposure time of 3 min.

### Viral inactivation of diagnostic specimens

Following the removal of the diagnostic specimen from the Ziploc bag, the bag was decontaminated with 5% Micro-Chem and placed into a small solid waste bin lined with a biohazard bag. Whole blood specimens collected in EDTA were transferred to a labeled 2 mL tube using a plastic transfer pipette. The used transfer pipette was filled with 5% Micro-Chem from the liquid waste bin and then discharged into that same bin. Likewise, the EDTA vacutainer tube was immersed in the liquid waste bin. Following the manufacturer’s protocol, a 200-μL pipette was used to transfer 100 μL of whole blood from the 2 mL tube to a labeled Xpert Ebola Assay Specimen Reagent Bottle containing 2.5 mL of virucidal guanidinium thiocyanate lysis reagent (Cepheid, Sunnyvale, CA, US) [12]. After mixing the blood by pipetting up-down, the pipette tip was filled with 5% Micro-Chem from the liquid waste bin and then discharged into that same bin. The remaining blood in the Wheaton tube was not discarded until the test results were reported. Oral swabs were transferred directly into a labeled Xpert Ebola Assay Specimen Reagent Bottle. Before closing the lid of the Specimen Reagent Bottle, the stem of the swab was broken-off by bending to one side. The stem and the original swab tube were immersed in the liquid waste bin containing 5% Micro-Chem. The swab specimen was then mixed with the contents of the Specimen Reagent Bottle by gentle inversion.

### Removal of the GeneXpert Sample Reagent Bottle from the RCK

The GeneXpert Sample Reagent Bottle (Cepheid), inner surface area and gloves of the RCK, as well as all items within the unit were surface decontaminated with 5% Micro-Chem prior to the removal of the Sample Reagent Bottle from the RCK. After opening the inner airlock on one of the small bag-in/bag out tubes, the decontaminated Sample Reagent Bottle was placed in the tube, the tube was saturated with 5% Micro-Chem and the inner airlock was closed. The Sample Reagent Bottle was incubated between the two airlocks for 20 minutes to allow sufficient time for complete viral inactivation of the diagnostic specimen. The outer airlock was then opened, the Sample Reagent Bottle was removed, and the outer airlock was closed.

### Preparation of the Xpert Ebola Assay Cartridge for testing

Donning a procedure-dedicated gown and single pair of gloves while in the GeneXpert preparation area, the Xpert Ebola Assay Cartridge was prepared according to the manufacturer’s instructions and placed into the GeneXpert IV Dx Instrument (four-module configuration; Cephid). The GeneXpert preparation area was then cleaned with fresh 0.5% chlorine. Following the completion of testing, the used Xpert Ebola Assay Cartridge was removed from the GeneXpert Dx Instrument and placed into the solid waste bin.

### Interpreting and reporting results

Upon testing completion, the specimen adequacy, specimen processing, and probe check controls were reviewed and the test results (Ebola glycoprotein [GP] and nucleoprotein [NP]) were only considered valid and reportable if the quality control (QC) checks passed. The QC and test results were manually recorded in the Specimen Log, electronically saved as a PDF, and printed for enclosure in the patient’s medical record. The test results were interpreted as presumptively positive for EBOV if either Ebola GP or Ebola NP were positive. The test results, patient demographic, and specimen timestamp information were securely emailed to the UVRI VHF Program (Entebbe), the ordering physician, Kasese District Health Officer and Surveillance Officer, and the Public Health Emergency Operations Center (PHEOC), with the following statement “GenXpert Results are considered presumptive and will be confirmed by the UVRI-VHF Laboratory”.

### Daily shut-down of RCK

After the test result was reported, the remaining whole blood specimen within the RCK was discarded by immersing the opened tube in the liquid waste bin containing 5% Micro-Chem. Prior to powering-off the air pump, the RCK’s inner surface area, gloves, and all the items within the unit were surface decontaminated with 5% Micro-Chem.

### Waste disposal

#### RCK waste

All full liquid and solid waste bins within the RCK were left overnight prior to processing the waste for removal. As the first task of the day, the decontaminated liquid contents of the liquid waste bin were carefully poured through a funnel into a leak-proof, lidded, plastic disposable container (e.g., used water bottle). The plastic disposable container was closed, placed into a biohazard bag, and then tied closed. After the remaining solid contents (i.e., transfer pipettes, pipette tips, EDTA vacutainers, oral swab tubes and 2 mL tubes) of the liquid waste bin were transferred to the solid waste bin, the biohazard bag lining the solid waste bin was removed, tied closed, surface decontaminated with 5% Micro-Chem, and then placed into a second biohazard that was tied closed. The bagged liquid and solid waste, as well as the entire inner surface area and contents of the RCK, were decontaminated with 5% Micro-Chem before the waste was removed from the bag-in/bag-out tube following the exact procedures described in the section “Movement of items in and out of the RCK”. Immediately after the solid and liquid waste was removed from the RCK, both bags were placed in a large biohazard bag that was tied closed and then decontaminated with 5% Micro-Chem.

#### Main laboratory waste

The small biohazard bag lining the small solid waste bin located in the GeneXpert preparation area was removed, tied closed, decontaminated with 5% Micro-Chem and placed into the large waste bin in the main laboratory. After the biohazard bag from the large waste bin was tied closed and decontaminated with 5% Micro-Chem, it was placed in a second biohazard bag, tied closed and decontaminated with 5% Micro-Chem.

#### Waste incineration

A laboratorian transported the decontaminated, RCK triple-contained waste and the main laboratory double-bagged waste to the entrance of the Bwera General Hospital ETU. A Hospital Hygienist then incinerated the laboratory waste in the ETU incinerator pit.

## Results

### Deployment to the Kasese District and assessment of a potential laboratory site

Shortly after the Ugandan Minister of Health requested that UVRI and CDC jointly establish a frontline Ebola field laboratory in Kasese District on June 14, 2019, UVRI and CDC laboratorians deployed to the district along with the pre-inventoried and packed laboratory equipment, supplies, and consumables (June 16). After meeting with the Kasese DHT and Bwera General Hospital Administration on June 17, the UVRI and CDC laboratorians assessed a space at the hospital that was being utilized to perform MUWRP-supported antimicrobial resistance testing activities. The assessment revealed that the space was ideal for the establishment of an Ebola Field Laboratory, as it was: 1) close to the ETU at Bwera General Hospital, 2) proximal to the Mpondwe Border Post, which is the busiest crossing point between DRC and Uganda, 3) of appropriate size (11 ft x 11 ft) to safely perform all laboratory operations, 4) accessible at all times to UVRI and CDC laboratorians to perform testing, 5) available for use, and 6) in a secure area that was routinely patrolled by security.

### Establishment of the UVRI-CDC Ebola Field Laboratory at Bwera General Hospital

The site of the UVRI-CDC Ebola Field Laboratory at Bwera General Hospital was prepared for operation from June 18 to 21 by: 1) removing extraneous items, 2) disinfecting surfaces with 0.5% chlorine, 3) fitting the laboratory with work benches and new electrical outlets, 4) securing an uninterruptable power supply (UPS) for the GeneXpert Instrument by tapping into the UPS of Bwera General Hospital’s Clinical Laboratory, 5) finalizing location-specific standard operating procedures (e.g., waste and specimen management plans; specimen remnants were inactivated after testing was complete and never kept overnight), 6) restricting access to UVRI-CDC Ebola Field Laboratory staff (i.e., only UVRI-CDC Ebola Field Laboratory staff possessed keys to the double-locked laboratory entrance, the outer hospital gate was locked at night, and the hospital was patrolled by a guard), and 7) setting-up the RCK and GeneXpert instrument. Prior to testing patient specimens using GeneXpert technology, we verified that the instrument and the current lot of Xpert Ebola Assay Cartridges were performing as expected by testing positive and negative EBOV RNA controls obtained from the UVRI VHF Laboratory.

### Laboratory operations

The laboratory accepted specimens collected from alert and suspect cases of EVD from June 22 through the end of August 7, 2019 (n = 46 days), 13 days after the EVD outbreak was declared over in Uganda (42 days: two EBOV incubation periods). During this time, the laboratory was staffed by three teams of two laboratorians, with one laboratorian originating from UVRI and the second from CDC. Although UVRI laboratorians from the VHF Program can independently operate the Ebola Field Laboratory, UVRI requested CDC’s assistance to staff the field laboratory as their VHF Laboratory in Entebbe was receiving an increased number of diagnostic VHF specimens from throughout Uganda and neighboring countries due to the EVD outbreak in the DRC. To ensure adequate staff were present to process these samples, rotating CDC Atlanta staff supported a rotation of staff from UVRI. Generally, one laboratorian performed viral inactivation of patient specimens, while the second laboratorian prepared the inactivated specimens for Ebola Xpert testing, recorded data, and reported test results. The turn-around-time from specimen receipt to result reporting was consistently < 3 hours.

### Patient population and laboratory outcomes

The Ebola Field Laboratory received patient specimens for rule-out of EVD on 63.0% (29/46) of its operational days. During this time, 76 specimens were tested for the presence of EBOV (mean: 1.7 specimens per day, range: 0–6); 56 of the 76 specimens represented initial collections from a suspect case. The majority of suspect case specimens collected (73.7%, 56/76) were drawn from patients shortly after they were admitted to the Bwera General Hospital ETU (89.3%, 50/56), Bwera General Hospital (1.8%, 1/56), or other health care facilities in western Uganda (7.1%, 4/56); in one instance a specimen was collected from a person that was reported by the community to have suddenly died (1.8%, 1/56). Most of the initial collections were whole blood specimens taken from live patients (91.1%, 51/56), while only a few were oral swabs taken from corpses soon after death (8.9%, 5/56). A minority of the specimens represented repeat collections (26.3%, 20/76) that were taken from patients whose initial specimens were collected <72 hours after symptom onset.

Patient information, clinical signs and symptoms, clinical status, epidemiological risk factors, and exposure information recorded on the CRF were used to assess the demographic composition of the patient population and recent travel history, as well as determine whether, or not, patients met the alert case, suspect case and/or the routine VHF surveillance case definition(s). Considering the data that was recorded on CRFs corresponding to initial specimen collections only (n = 56), the mean age of the patient population ranged from 0.5–70.0 years (mean: 23.7 years). Approximately half of the patients were male (48.2%, 27/56) and the majority reported that they had traveled to the DRC within the past month (58.9%, 33/56). Of these 56 patients, 83.9% (47/56) met the EVD alert case definition, 69.6% (39/56) met the EVD suspect case definition, and 48.2% (27/56) met the more stringent VHF suspect case definition used by the VHF Surveillance and Laboratory Program at UVRI for routine VHF surveillance (Table 2).

All 76 of the specimens tested negative for EBOV by the Xpert Ebola Assay and confirmatory testing of duplicate specimens by the VHF Surveillance and Laboratory Program at the UVRI confirmed that all specimens were negative for EBOV, as well as other hemorrhagic fever viruses known to circulate in Uganda.

## Discussion

The UVRI-CDC Ebola Field Laboratory played an important role in patient triage and management, and epidemiological surveillance of suspect cases of EVD by providing diagnostic results in <3 hours. Although the VHF Surveillance and Laboratory Program at UVRI in Entebbe routinely tests specimens collected from suspect VHF cases for EBOV, it is a 7–8-hour drive from the Kasese District. Depending on the time-of-day specimens are collected, the turnaround time for specimens referred from distant districts can be >24 hours.

As part of the 2018 EVD preparedness activities in Uganda, UVRI and CDC laboratorians assembled four trunks, measuring up to 4.33 ft^3^, that contained GeneXpert and RCK equipment, as well as enough laboratory supplies to operate a frontline Ebola field laboratory safely and securely for >1 month. Owing to this preparation, staff and supplies were able to rapidly deploy to the Kasese District to establish an Ebola field laboratory. The ease of setting-up and operating the UVRI-CDC Ebola Field Laboratory at Bwera General Hospital was facilitated by the field laboratory’s simplistic specimen collection and diagnostic algorithm. Utilization of the RCK for inactivation of blood and oral swab specimens and the Xpert Ebola Assay for EBOV testing (integrated nucleic acid extraction and qRT-PCR) required only a single laboratory room with two designated workspaces. In contrast, high-throughput VHF field laboratories such as the CDC Ebola Field Laboratory that was established in Sierra Leone during the 2014–2015 West African EVD Outbreak [13], typically require an outdoor workspace for specimen inactivation while donning full PPE and three separated indoor workspaces for: 1) the preparation of clean nucleic acid extraction reagents and qRT-PCR master mixes, 2) RNA extraction, and 3) the addition of RNA template to the qRT-PCR master mix. Electrical power at the field laboratory was only required when operating the GeneXpert Instrument, as the exhaust air pump for the RCK was battery-powered and the Xpert Ebola Assay cartridges did not require refrigeration. Furthermore, refrigeration of specimens collected for testing at the field laboratory was not needed, as they were immediately tested and then discarded. The cost of Xpert Ebola Assay cartridges (~$20 USD/cartridge) and a GeneXpert instrument (~$17,000 USD) is similar to the cost of kit-based EBOV qRT-PCR assays (e.g., US CDC Ebola Assay) and a qRT-PCR instrument (e.g., Bio-Rad CFX96 Touch Real-Time PCR Detection System) [14]. Additionally, having the laboratory on-site 1) built trust with non-governmental organizations (e.g., Médecins Sans Frontières) and the 2) local community. Rather than having to wait up to 24 hours for an EBOV test result, the Bwera General Hospital ETU could recieve a presumptive test result within three hours of sample collection and families with concerned loved ones could have relief from the fear and anxiety shortly after. Given that the cost of operating a targeted field laboratory for the defined period was not more expensive than the cost of sample transport and testing, the gain achieved from points 1 and 2 made deploying a field laboratory an intelligent investment.

Although the two-person field laboratory team was able to easily process and test patient specimens using the RCK and four-module GeneXpert IV Dx Instrument, we received a maximum of six specimens on a single day. Using this field laboratory set-up and considering a 12-hour work shift, we estimate that the maximum specimen throughput would be 44 specimens (Xpert Ebola Assay run-time is 90 minutes). Larger EVD outbreak responses requiring an increased specimen throughput may want to consider using a 16-module GeneXpert Dx instrument, and additional RCKs and laboratory staff. Alternatively, if dependable electricity, highly trained laboratory staff, and sufficient space are available, standing-up a high-throughput field laboratory, such as the CDC Ebola Field Laboratory that was established in Sierra Leone during the 2014–2015 West African EVD Outbreak [13], should be considered. During this deployment of the field laboratory, we tapped into the hospital clinical laboratory’s UPS to secure electricity for the GeneXpert Instrument. Field laboratories situated in more rural locations may require the deployment of an independent electricity source, such as a gas-powered generator with a regulator.

UVRI is designated as the national VHF Reference Laboratory. Therefore, the collection and transportation of a duplicate patient specimen to UVRI was critical in preserving the structure of the national VHF reference laboratory system, confirming all negative Xpert Ebola Assay results, and ensuring that each patient specimen was tested for other hemorrhagic fever viruses that circulate in Uganda. From the time the VHF Surveillance and Laboratory Program at UVRI was established in 2010 [9] until the end of December 2019, it has confirmed 6 independent filovirus outbreaks in Uganda due to infection with SUDV (n = 3) [15–17] and the marburgviruses (Marburg and Ravn viruses; n = 3) [8,18–20]. The VHF Program also detected 32 CCHF human cases and 41 RVF human cases during this same time period [21]. Of notable importance, 10 of these RVF cases and 18 of the CCHF cases were detected after the EVD outbreak was declared in the DRC in August 2019. Prior to 2010, Uganda also experienced outbreaks due to infection with BDBV [22,23], as well as additional outbreaks due to infection with SUDV [24], MARV and RAVV [25–27]. Notably, a case of RVF in Kasese District was confirmed by the VHF Laboratory one week prior to the confirmation of this imported outbreak of EVD. These data underscore the importance of ensuring that all specimens collected during a VHF outbreak in Uganda are promptly transferred to the VHF Laboratory at UVRI to rule-out infection with other hemorrhagic fever viruses that circulate in Uganda. This will help ensure the timely detection of VHF cases and prevent person-to-person virus transmission within the community.

The UVRI-CDC Ebola Field Laboratory at Bwera General Hospital discontinued operations 13 days after the imported EVD outbreak was declared over in Uganda. Following the transport of laboratory equipment, supplies, and consumables back to UVRI, the consumables were inventoried and replenished to initial deployment quantities. On August 28, 2019, a 9-year-old child with EVD signs and symptoms was identified by screeners at the Mpondwe Border Post at the DRC-Uganda border and promptly transferred to the Bwera General Hospital ETU. A blood specimen from the child was sent to the VHF Surveillance and Laboratory Program at UVRI and test results released the next morning confirmed that the 9-year-old was positive for EBOV [28]. Due to the preparatory activities upon return from its initial mobilization, the UVRI-CDC Ebola Field Laboratory rapidly deployed back to Bwera General Hospital and began testing specimens from alert and suspect cases of EVD on August 31, 2019. During its second deployment, the field laboratory not only continued to be an integral part of the EVD Outbreak Response by supporting patient management and epidemiological surveillance, but was able to enhance clinical care by providing comprehensive metabolic panel results on all patients admitted to the ETU using a Piccolo Xpress Chemistry Analyzer contained within a second RCK. No further EVD cases were detected during this second deployment and the laboratory was demobilized after 72 days.

In summary, we recommend that others charged with providing laboratory support during an EVD outbreak in a remote area adapt a field laboratory strategy similar to the one described here. Due to the simplistic nature of our Ebola Field Laboratory approach, we were able to rapidly transport all necessary laboratory equipment and supplies in one vehicle to the field site, set-up the laboratory in a single room, and begin testing specimens from alert and suspect cases of EVD. This approach, together with our proximity to the ETU, allowed us to consistently provide EBOV diagnostic results within 3 hours of specimen receipt and meet requirements under International Health Regulations (2005) for the timely reporting of diagnostic results (<48 hours) during a public health emergency [29].
